# Successful treatment of pulmonary inflammatory myofibroblastic tumor with platinum‐pemetrexed: The first report of two cases

**DOI:** 10.1111/1759-7714.13520

**Published:** 2020-06-03

**Authors:** Xiaoyan Si, Hanping Wang, Xiaotong Zhang, Mengzhao Wang, Yan You, Li Zhang

**Affiliations:** ^1^ Department of Pulmonary and Critical Care Medicine Peking Union Medical College Hospital Beijing China; ^2^ Department of Pathology Peking Union Medical College Hospital Beijing China

**Keywords:** Inflammatory myofibroblastic tumor, pemetrexed, platinum

## Abstract

Pulmonary inflammatory myofibroblastic tumor (IMT) is a rare tumor. Here, we report two cases of pulmonary IMT successfully treated with platinum and pemetrexed. The results from this study suggest that platinum‐pemetrexed might be an effective therapy in patients with IMT, but requires further investigation.

## Introduction

Inflammatory myofibroblastic tumor (IMT) is a rare mesenchymal tumor. It can arise in the soft tissue of almost every organ. IMTs are characterized by spindle cell proliferation with an inflammatory infiltrate composed of lymphocytes, plasma cells, and eosinophils.^1^ Complete surgical resection is the most effective treatment for surgically accessible IMT. However, systemic therapies should be considered for inoperable IMT. Gene alterations of *ALK*, *ROS1*, *NTRK*, *RET*, and *PDGFRβ* have been detected in some patients with IMT, and targeted drugs are available for patients harboring those rearrangements.^2^, ^3^ Nonsteroidal anti‐inflammatory drugs (NSAIDs), steroids, and chemotherapy have been reported to treat IMT.^4^ However, little evidence is available on the efficacy of chemotherapy for IMT.^5^ Here, we report two cases of IMT treated successfully with platinum and pemetrexed.

### Case report

### Case 1

**Figure 1:**
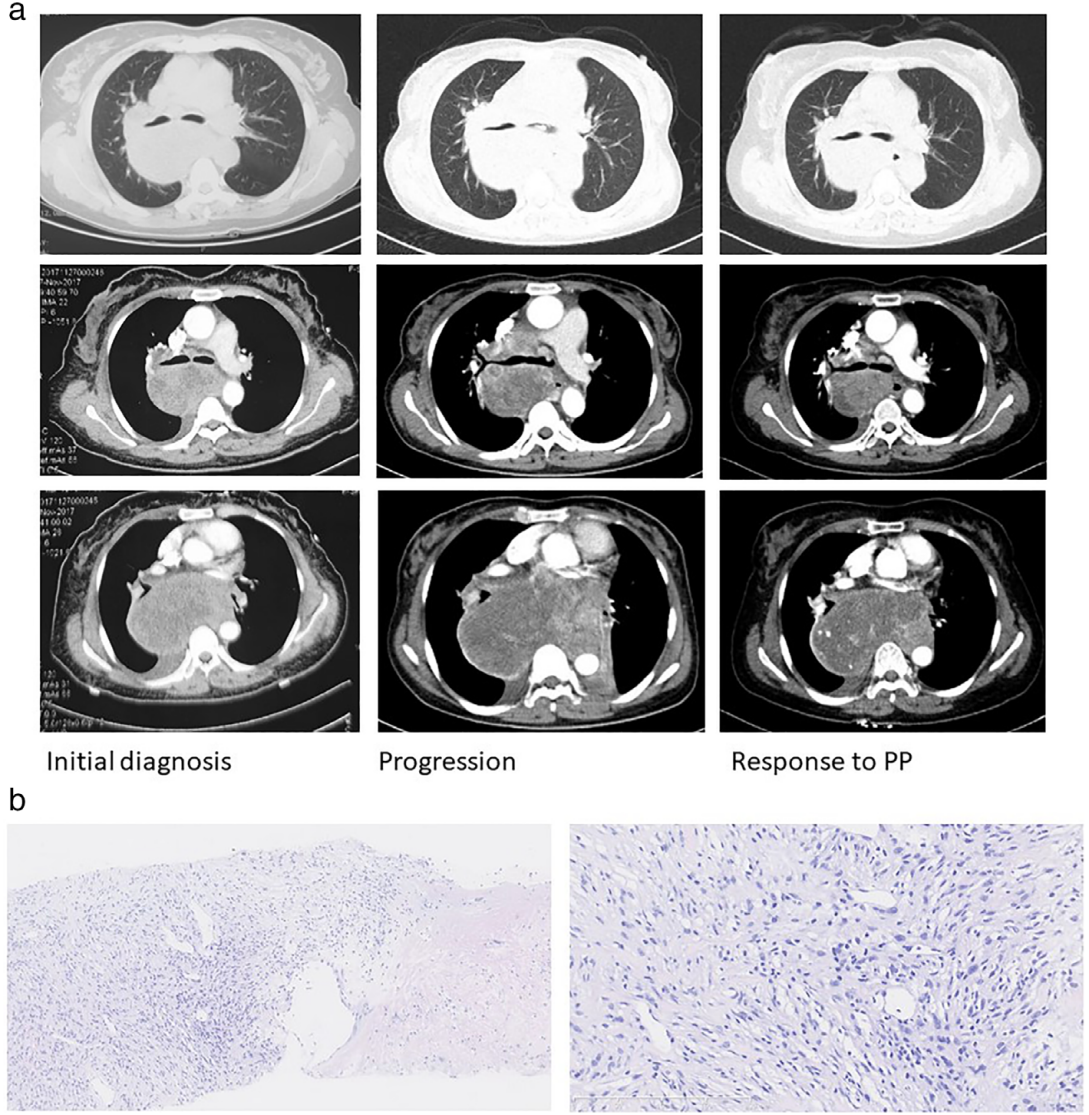
Case 1. Imaging and pathology. (**a**) The computed tomography (CT) scan of the chest showed a mass in the posterior mediastinum on initial diagnosis. The left main bronchus was involved when the disease progressed. The lesion in the left main bronchus responded to treatment with pemetrexed and carboplatin. (**b**) Pathologic findings from the lesion in the posterior mediastinum demonstrated a spindle cell neoplasm with inflammatory cell infiltration. (**a**) hematoxylin and eosin stain ×50; (**b**) hematoxylin and eosin stain ×200.

A 46‐year‐old woman presented with a history of worsening dyspnea on exertion and a dry cough. The computed tomography (CT) scan of the chest showed a mass in the posterior mediastinum. Analysis of tissue specimens obtained by CT‐guided percutaneous puncture demonstrated an abundant spindle cell neoplasm with inflammatory cell infiltration. The immunohistochemical stain was positive for BCL‐2 and negative for S100, CD34, CD117, and ALK‐D5F3. The histologic and immunostaining results suggested an inflammatory myofibroblastic tumor (IMT). Next‐generation sequencing (NGS) of plasma did not detect any alteration in ALK, ROS1, NTRK, or RET. The symptoms of dyspnea progressed, and the CT scan demonstrated an intraluminal lesion in the left main bronchus. Methylprednisolone 40 mg was given for 10 days, but dyspnea continued. She subsequently received pemetrexed (500 mg/m^2^) and carboplatin (area under curve 5) once every three weeks for four cycles, then maintenance therapy with pemetrexed (500mg/m^2^) once every three weeks. After one cycle of chemotherapy, the dyspnea was relieved, and performance status improved significantly (The Eastern Cooperative Oncology Group Performance Status from 4 to 0). A follow‐up CT scan showed remission of the intraluminal lesion in the left main bronchus (Fig 1). Progression‐free survival (PFS) was 15 months at the time of submission of this study.

### Case 2

**Figure 2:**
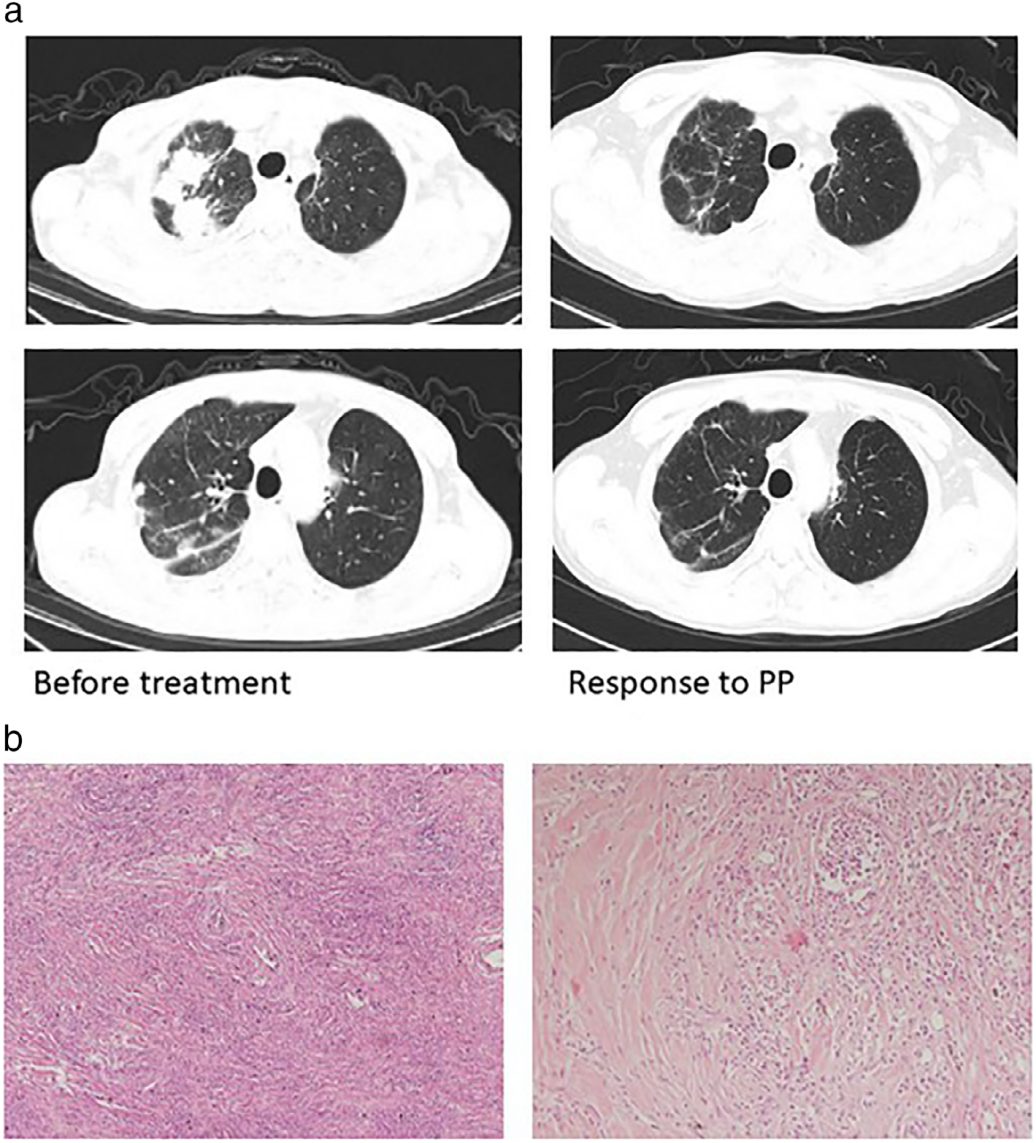
Case 2. Imaging and pathology. (**a**) The computed tomography (CT) scan of the chest showed lesions in the upper lobe of the right lung. The lesions responded to treatment with pemetrexed and cisplatin. (**b**) Pathologic findings from the upper lobe of the right lung demonstrated a spindle cell neoplasm with inflammatory cell infiltration. (**a**) hematoxylin and eosin stain ×40; (**b**) hematoxylin and eosin stain ×100.

A 50‐year‐old man with a past medical history of intestinal tuberculosis presented with intermittent fever since 2012. The CT scan of the chest showed a mass in the left lung. The fever subsided after left lung lesion resection. Pathology revealed IMT. Fever recurred in August 2017, with accompanying pain in the right maxillary sinus. The CT scan showed a mass in the right maxillary sinus with involvement of adjacent bone. Biopsy of the right maxillary sinus showed inflammation. Considering the possibility of tuberculosis, isoniazid, rifampicin, ethambutol, and pyrazinamide was given for three months. However, the patient’s symptoms did not improve. The CT scan of his chest showed a mass in the upper lobe of the right lung in April 2018. Biopsy of the right lung showed IMT which was negative for ALK. He received pemetrexed (500 mg/m^2^) and cisplatin (60 mg/m^2^) once every three weeks for six cycles, and then maintenance therapy with pemetrexed (500 mg/m^2^) once every three weeks. The lesions in the right lung indicated disease remission. (Fig 2) PFS was 13 months at the time of submission of this study.

## Discussion

IMT is a neoplasm of intermediate biological potential, which may recur and infrequently metastasizes. Approximately half of IMTs contain translocations of the *ALK* gene, and ALK inhibitors crizotinib and ceretinib are effective in those patients.^6^, ^7^ There is no standard treatment for inoperable IMT without driver gene alteration due to the rarity of IMT. Various cytotoxic agents or regimens, including methotrexate, vinorelbine, vincristine, cyclophosphamide, doxorubicin, 5‐fluorouracil, cisplatin, carboplatin, paclitaxel, ifosfamide, and etoposide have been reported to treat IMT. Methotrexate, an antifolate, is one of the most frequently reported agents for IMT.^5^ Pemetrexed is an antifolate, which is structurally similar to methotrexate. It inhibits multiple folate‐dependent enzymes responsible for tumor growth, including thymidylate synthase, dihydrofolate reductase, and glycinamide ribonucleotide formyltransferase. Pemetrexed is used to treat nonsquamous non‐small cell lung cancer (NSCLC) and mesothelioma. Moreover, studies have demonstrated fewer adverse events and better tolerance for patients treated with pemetrexed. Pemetrexed is even used in the maintenance setting because of its efficacy and low cumulative toxicity.^8^ Considering the poor performance status of the two patients in this study, we chose pemetrexed and platinum and obtained written informed consent from patients for off‐label use. The two patients did not experience any severe toxicities.

There were several limitations in this study. First, premedication regimens of dexamethasone 3.75 mg twice daily for three days were given with pemetrexed‐based therapy. Therefore, the effect of corticosteroid on IMT could not be completely excluded. Before the administration of pemetrexed, both patients had no response to steroids. During treatment of pemetrexed, the steroid was given for three days every cycle, but we did not consider it enough to control the tumor. Therefore, tumor shrinkage was attributed to pemetrexed and platinum therapy. Second, NGS of tissue was not conducted due to inadequate specimens in Case 1. Although NGS of plasma did not detect the targeted gene, the possibility of a false‐negative result should be considered. Third, the optimal course of maintenance treatment was not determined.

To the best of our knowledge, this is the first report on the efficacy of platinum‐pemetrexed in IMT. Platinum‐pemetrexed might be an effective therapy in patients with IMT, but this requires further investigation.

## Disclosure

The authors have no potential conflicts of interest to disclose.
